# Transient Nutrient Deficiencies in Pea: Consequences on Nutrient Uptake, Remobilization, and Seed Quality

**DOI:** 10.3389/fpls.2021.785221

**Published:** 2021-12-23

**Authors:** Cécile Jacques, Marion Forest, Vincent Durey, Christophe Salon, Alain Ourry, Marion Prudent

**Affiliations:** ^1^Agroécologie, AgroSup Dijon, INRAE, Université de Bourgogne, University Bourgogne Franche-Comté, Dijon, France; ^2^UMR 950 Ecophysiologie Végétale, Agronomie et Nutritions N, C, S, INRAE, Normandie Université, UNICAEN, Caen, France

**Keywords:** grain legume, ionome, nutrient deficiency, nutrient interactions, seed mineral composition, agroecology

## Abstract

Legume plants, such as peas, are of significant nutritional interest for both humans and animals. However, plant nutrition and thus, seed composition, depends on soil mineral nutrient availability. Understanding the impact of their deprivation on the plant mineral nutrient content, net uptake, and remobilization is of key importance but remains complex as the elements of the plant ionome are linked in intricate networks, one element deprivation impacting uptake and remobilization of other nutrients. To get a better insight into pea mineral nutrition, the transitory deprivations of 13 mineral nutrients were imposed during the vegetative growth phase. Thereafter, plants were grown under optimal mineral conditions until physiological maturity. Plant nutritional status and seed quality impacts caused by the deprivations were characterized using measurement of mineral nutrient concentration and plant biomass allocation. Our results highlight: (i) the preferential allocation of dry weight and elements to shoots at the expense of the roots under non-limiting conditions, and more particularly to the tendrils in comparison to the other shoot organs, (ii) the positive and/or negative impact of one mineral nutrient deprivation on other elements of the ionome, (iii) four different remobilization strategies for eight mineral nutrients, and (iv) possible strategies to improve seed quality *via* fine control of fertilization during a period of mineral nutrient deficiency.

## Introduction

Plants are sessile organisms that must constantly adapt to fluctuating local conditions. Their growth and development depend on water and mineral nutrient availability around the root system that can be modulated in time and space by soil physicochemical properties, such as rhizospheric soil pH (Verma and Minhas, 1987) that results from interactions between the chemical form of the element, root rhizodeposition, and the activity of microbial communities. Abiotic factors including extreme temperatures, anaerobic conditions, or low water availability can also impair the mineral nutrient uptake by the plant. For example, soil moisture influences mineral nutrient mobility in the soil solution (Wiersum, 1958; Barber, 1962). The decrease of nutrient mobility in the soil limits the movement of elements from the soil solution to the rhizosphere and thus the availability of the mineral nutrients for the plant. The plant element contents have been defined as the plant ionome that can be subdivided into two categories, essential elements (or nutrients) and beneficial elements. Essential elements constitute the functional ionome and correspond to those that are needed to complete the plant lifecycle, are not replaceable by any other element to ensure a biological function, and are directly involved in plant metabolism (Arnon and Stout, 1939). Essential elements are divided into two groups, macro-nutrients, and micro-nutrients. Macro-nutrients include carbon (C), hydrogen (H), oxygen (O), nitrogen (N), phosphorus (P), potassium (K), calcium (Ca), sulfur (S), and magnesium (Mg), while micro-nutrients include iron (Fe), copper (Cu), zinc (Zn), manganese (Mn), molybdenum (Mo), bore (B), chlorine (Cl), and nickel (Ni). These two groups of nutrients differ in the quantity needed for optimal plant growth. Indeed, macro-nutrient needs for plants represent more than 0.1% of the plant dry weight while for micro-nutrients, this represents less than 0.1% of the plant dry weight (Kirkby, 2012; Maillard et al., 2015). Beneficial elements comprise vanadium (V), cobalt (Co), or sodium (Na), and present positive effects on growth under particular growth conditions and differ among species (Kirkby, 2012; Maillard et al., 2015).

The uptake of these mineral nutrients can be modulated either *via*: (i) mechanisms triggering the root system morphological adaptations, (ii) regulations of the element transporter activities of the roots or storage of elements within plants, or (iii) rhizosphere modifications. For example, some nutrient deficiencies induce root system plasticity to improve soil prospection (López-Bucio et al., 2003; Gruber et al., 2013; Giehl and von Wiren, 2014). For instance, P or Fe deficiency increases root hair density in *Arabidopsis thaliana* (Ma et al., 2001; López-Bucio et al., 2003). N deficiency may increase primary root and lateral root length (López-Bucio et al., 2003). Mineral nutrient uptake is controlled by transporter activities which select the elements necessary for optimal plant growth, depending on their availability (Gojon et al., 2009; Tejada-Jiménez et al., 2009; Llamas et al., 2011). Soil mineral nutrients absorbed by roots can then be stored or remobilized in different plant compartments, to optimize nutrient availability for growing tissues. In this way, their storage duration during the vegetative stage depends on the need and on the availability of mineral nutrients to support the growth of new tissues (Malagoli, 2005; Abdallah et al., 2010; White, 2012) while during the reproductive stage, root activity and mineral nutrient uptake are reduced (Malagoli, 2005). Alternatively, storing the element in plant compartments such as vacuoles can reduce their overall potential toxicity for plants (Pittman, 2005; Millaleo et al., 2010).

Uptake, storage, and remobilization, all of which depend on the species, nutrient availability, and environmental conditions, are three important processes to ensure the optimal growth of plants and thus the yield and quality of seeds. Under mineral nutrient deficiency or during the reproductive phase, the exportation of elements from old tissues to growing tissues plays a crucial role in optimal growth and yield (Masclaux-Daubresse et al., 2008). The remobilization rate is specific to each element of the plant ionome. Macro-nutrients (except Ca) are rapidly transported by phloem in comparison to micro-nutrients (White, 2012). As such, it has been reported in three legume species (pea, white lupin, and narrow-leaved lupin), that N, P, and K present efficiency of mobilization of 60 to 90% from senescing organs to seeds whereas that of Mg, Zn, Mn, Fe, and Cu is lower, between 20 and 60 % (Hocking and Pate, 1977; Maillard et al., 2015). Moreover, Ca and Mn are minimally mobile in the phloem but remobilized from the root *via* xylem (Biddulph et al., 1959; White, 2012; Maillard et al., 2015). In *A. thaliana*, during seed filling, 48% of K is remobilized from leaves but less than 30% for Fe, P, S, Zn, and Cu, whereas Mg, Ca, and Mn are not remobilized (Waters and Grusak, 2008). The characterization of the mechanisms involved in these processes is therefore required to improve our knowledge of plant needs according to species, environmental conditions, and considered stages.

The elements that compose the plant ionome are linked together by a complex network extending from the soil to plant tissues (Lahner et al., 2003; Salt et al., 2008; Baxter, 2015). First, some mineral nutrients are taken up by common transporters, such as for S and Mo (Dudev and Lim, 2004), K and Na (Gassmann et al., 1996), and divalent metals (Pilon et al., 2009). This can induce competition between mineral nutrients for their uptake (Gassmann et al., 1996; Dudev and Lim, 2004; Alhendawi et al., 2005; Pilon et al., 2009). For instance, the divalent metal co-transporter IRT1 which has a high affinity for Fe is up-regulated by Fe deficiency and other cations (Ni^2+^, Cu^2+^, Mn^2+^, Zn^2+^) are then also increasingly taken up (Pilon et al., 2009). Second, some elements share similar roles in biological processes. Plant homeostasis results from interactions among mineral elements (Kirkby and Knight, 1977; Sorin et al., 2015). To maintain osmotic and acido-basic equilibrium under conditions of deficiency or overaccumulation, some mineral nutrients may be increasingly taken up under a non-optimal total ionic charge. For instance, a S deficiency induces a disequilibrium of negative ionic charge compensated for by an increase of NO_3_^–^ and PO_4_^2–^ uptake (Sorin et al., 2015). In contrast, an increase in the quantity of NO_3_^–^ in plant tissues induces an increase in K^+^, Ca^2+^, Mg^2+^, and Na^+^ uptake to compensate for the negative charge (Kirkby and Knight, 1977). Biosynthetic pathways requiring various mineral nutrients can also be impacted by the deficiency of one of these elements. For instance, the pathway of Mo cofactor biosynthesis involves Cu and Zn (Kuper et al., 2004; Schwarz and Mendel, 2006; Llamas et al., 2011). Thus, Cu or Zn deficiency may have a negative impact on the Mo consumption in this pathway.

As such, due to different interactions between elements, the impact of an elementary deprivation on plant growth and yield is the result of a complex network and cannot be reduced to a simple deficiency of the missing element.

Thanks to the smaller size of the ionome dataset as compared with the transcriptomic and metabolomic dataset, recent studies of the ionome have provided new insights into the characterization of plant nutritional status (Baxter et al., 2008). The negative impacts of elementary deficiencies on seed quantity and quality have been recognized for years (McGrath and Zhao, 1996). For instance, B deficiency in canola plants negatively impacts plant yields (Grant and Bailey, 1993) while S deficiency reduces the accumulation of proteins rich in S in seeds in rapeseed and pea plants (D’Hooghe et al., 2014; Henriet et al., 2019). However, the effects of transitory deficiencies, i.e., during a certain period of the crop cycle, on seed quality are not well established. In some cases, the deficiency in one element could serve to enhance the rate of other elements during vegetative growth, as previously reported for Fe deficiency and other metal cations (Pilon et al., 2009). So, it can be hypothesized that a higher metal cation in vegetative tissues may increase their remobilization to the seeds. Such impacts on seed composition can be beneficial for food quality under the context of human food deficiencies in Fe, Mg, Cu, and Zn throughout the world (Fan et al., 2008). Pea seeds are rich in Zn and Fe and can provide a solution to the most important micro-nutrient deficiencies (Amarakoon et al., 2012). Thus, in a species whose consumption is recommended by nutritionists, the characterization of seed mineral content after transient nutrient deficiencies could help in targeting the appropriate fertilization.

To extend our understanding of pea nutrition and response to mineral nutrient deprivation, several issues have been examined in this article. Firstly, elements were classified in terms of their quantity needed for plant optimal growth and their allocation into four different organs of the pea plant (roots, stems, stipules, tendrils). Secondly, we analyzed the ionome composition under nutrient deprivation to confirm interactions observed in other species and to characterize the specific interactions related to the pea plant. Moreover, this characterization allowed us to obtain an ionomic imprint of the different deprivations. Thirdly, an analysis of mature seeds enabled us to identify the effects of each transient deprivation on yield and seed quality at physiological maturity.

## Materials and Methods

### Plant Growth Under Hydroponic Conditions

**FIGURE 1:**
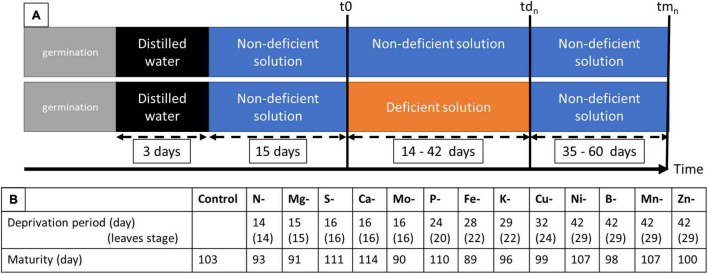
Experimental design **(A)** showing the duration of deprivation period between t0 and td_*n*_ and life cycle to maturity between t0 and tm_*n.*_ **(B)** Control corresponds to the solution needed for optimal growth; while solutions deficient in one element concern N, S, P, K, Ca, Mg, Cu, Ni, Mo, B, Zn, Mn, and Fe; t0 correspond to the first sample of the control plant before deprivation (7 pots of 2 plants each in control solution); td_*n*_ correspond to the sample at deficiency establishment that depends on the element considered (6 pots of 2 plants each of control and deficiency solutions); tm_*n*_ correspond to the sample at maturity that depends on the element considered (3 pots of 2 plants each).

Pea seeds (Pisum sativum L. cv. Kayanne, obtained from KWS Momont, Mons-en-Pévèle, France) were calibrated, surface sterilized by exposure to 70% ethanol for 1 min, then to 0.6% sodium hypochlorite for 10 min. The seeds were then imbibed in distilled water for 2 h and pre-germinated in trays containing sand at 8% humidity for three days in the dark at 22°C, in a Fitoclima S600 germinator (Aralab, Rio de Mouro, Portugal). Seedlings were then transferred to the greenhouse in a 30 L container filled with demineralized water over 3 days, to favor radicle length and lead to homogenized seedling growth. The greenhouse environmental conditions were 21.3 ± 1.7°C during the day and 16.5 ± 1.0°C at night, with a photoperiod of 16h with artificial lighting (MACS 400W; Mazda, Dijon, France), allowing for a mean of 188.12 μmol/m^2^/s. The seedlings were then transferred to 208 5 L pots (two seedlings per pot) containing a nutritive solution hereafter referred to as “Control solution, C” described in Table 1. After 15 days of plant growth under plethoric mineral nutrition (t0), pots were divided into 14 groups with one “Control” and 13 “Deficient” solutions (Figure 1A) whose compositions are detailed in Table 1. The 13 elementary deprived solutions used include N deficiency (N-), S deficiency (S-), P deficiency (P-), K deficiency (K-), Ca deficiency (Ca-), Cu deficiency (Cu-), Ni deficiency (Ni-), Mo deficiency (Mo-), B deficiency (B-), Zn deficiency (Zn-), Mg deficiency (Mg-), Mn deficiency (Mn-), and Fe deficiency (Fe-) (Figure 1A). All elements that composed each of the 14 nutrient solutions were added in excess, to avoid any potential competition for elements between the two plants of the pot. At the beginning of the plant nutrient deficiency period (t0) (Figure 1A), seven pots were harvested, corresponding to 14 plants. On the 201 remaining pots, a mark with attached twines identified organs formed before t0 from organs formed after t0. Three times a week, the plants were removed from their pots, weighed, and replaced in their pots. As soon as a growth cessation was observed for plants grown under a nutrient (n) deprivation, the plants in this group were harvested (td_n_) along with plants from the control group (Control). The duration of the nutrient deprivation period thus depended on the nutrient that had been depleted from the solution (Figure 1B). When no growth cessation was observed, plants were arbitrarily harvested at td_n_ = 42 days after nutrient deprivation imposition. At each td_n_, 6 deficient pots (12 plants) and 6 control pots (12 plants) were harvested. Their root surface was rinsed with osmotic water and plants were separated into seven samples: non-nodulated roots, stems, stipules, and tendrils formed before nutrient deprivation at t0 (hereafter referred to as stembf, stipulebf, and tendrilbf, respectively) and the stems, stipules, and tendrils developed after t0 (hereafter called stemaf, stipuleaf, and tendrilaf, respectively). At the end of the deficiency period (td_n_), three pots per “Deficient” solution were filled with the non-deficient “Control” solution until the plants reached their physiological maturity stage (tm_n_) (Figure 1A). The time to reach the physiological maturity phase depended on the deficiency considered (Figure 1B). At tm_n_, only seeds were harvested for further analysis and counted to determine yield components.

**TABLE 1:** Composition of control and nutrient-deprived solutions.

	Nutrient solutions													
Salt concentration	Control	N-	K-	Ca-	P-	S-	Mg-	Fe-	B-	Mn-	Zn-	Cu-	Mo-	Ni-
CaCO_3_	2000	3250	5000	0	2000	2000	2000	2000	2000	2000	2000	2000	2000	2000
KNO_3_	1250	0	0	3750	1250	1250	1250	1250	1250	1250	1250	1250	1250	1250
Ca(NO_3_)2, 4H_2_O	1250	0	1875	0	1250	1250	1250	1250	1250	1250	1250	1250	1250	1250
KH_2_PO_4_	250	250	0	250	0	250	250	250	250	250	250	250	250	250
MgSO_4_	500	500	500	500	500	0	0	500	500	500	500	500	500	500
H_3_BO_3_	10	10	10	10	10	10	10	10	0	10	10	10	10	10
CuSO_4_	0.7	0.7	0.7	0.7	0.7	0.7	0.7	0.7	0.7	0.7	0.7	0	0.7	0.7
CoCl_2_	0.1	0.1	0.1	0.1	0.1	0.1	0.1	0.1	0.1	0.1	0.1	0.1	0.1	0.1
NiCl_2_	0.04	0.04	0.04	0.04	0.04	0.04	0.04	0.04	0.04	0.04	0.04	0.04	0.04	0
SiO_2_	100	100	100	100	100	100	100	100	100	100	100	100	100	100
Na_2_O	38.78	38.78	38.78	38.78	38.78	38.78	38.78	38.78	38.78	38.78	38.78	38.78	38.78	38.78
Na_2_SO_4_	0	0	0	0	0	0	0	0	0	5	3	0.7	0	0
CaCl_2_,2 H2O	1250	1250	625	0	1250	1250	1250	1250	1250	1250	1250	1250	1250	1250
KCl	250	250	0	2000	250	0	250	250	250	250	250	250	250	250
HCl	0	0	1500	0	0	0	0	500	0	0	0	0	0	0
NaOH	0	0	0	0	0	0	0	400	0	0	0	0	0	0
KOH	0	1250	0	50	250	0	0	0	0	0	0	0	0	0
KHSO_4_	0	0	0	0	0	0	500	0	0	0	0	0	0	0
MnSO_4_,4H_2_O	5	5	5	5	5	5	5	5	5	0	5	5	5	5
ZnSO_4_,7H_2_O	3	3	3	3	3	3	3	3	3	3	0	3	3	3
(NH_4_)6 Mo_7_O_24_; 4H_2_O	0.7	0.7	0.7	0.7	0.7	0.7	0.7	0.7	0.7	0.7	0.7	0.7	0	0.7
MgCl_2_,6H_2_O	0	0	0	0	0	300	0	0	0	0	0	0	0	0
EDTA, (NaFe), 0.05% H_2_O	200	200	200	200	200	200	200	0	200	200	200	200	200	200
H_3_PO_4_	0	0	250	0	0	0	0	0	0	0	0	0	0	0
NH_4_NO_3_	3500	0	3500	3500	3500	3500	3500	3500	3500	3500	3500	3500	3500	3500

*Deprivation applied are N-, Nitrogen deprivation; K-, Potassium deprivation; Ca-, Calcium deprivation; P-, Phosphorus deprivation; S-, Sulfur deprivation; Mg-, Magnesium deprivation; Fe-, Iron deprivation; Mn-, Manganese deprivation; Mo-, Molybdenum deprivation; Zn-, Zinc deprivation; B-, Bore deprivation; Cu-, Copper deprivation; Ni-, Nickel deprivation. Salt concentration is given in μmol.L^–1^.*

### Elemental Analysis of Plant Tissues

Following each harvest, tissue samples including non-nodulated roots, stems, stipules, tendrils formed before nutrient deprivation (at t0 and td_n_) or after nutrient deprivation (at td_n_), or seeds at (tm_n_) were dried at 80°C for 48h, individually weighed to measure their dry weight and ground to a fine powder using MM 400 vibratory mixer mill (Retch, France). C and N concentrations were measured by Dumas procedure (Thermo electron NC 2500, Courtaboeuf, France). The other element concentrations [Sulphur (S), Phosphorus (P), Potassium (K), Calcium (Ca), Magnesium (Mg), Copper (Cu), Nickel (Ni), Manganese (Mn), Bore (B), Zinc (Zn), Iron (Fe), Vanadium (V), Cobalt (Co), Sodium (Na), Molybdenum (Mo)] were measured with a high resolution inductively coupled plasma mass spectrometer (HR ICP-MS, Element2, Thermo Fisher, Caen), following the methods previously described in Maillard et al. (2015).

### Calculations and Statistical Analyses

From the elemental measurements of tissues at t0, tdn_n_, and tm_n_, different variables were calculated, where td_n_ corresponds to the sample at the end of the deprivation period for element n, and tm_n_ corresponds to the sample at physiological maturity for element n.

The quantity (*Qty*) of element *n* in the tissue *x* (expressed in mg) was calculated as:


$$Q\,t\,y_{n\,\left(x\right)}={\left[n\right]}_{x}\,\text{×}\,D\,W_{x}$$

where [n]_*x*_ is the concentration of element n in the tissue x (in mg/g), and DW_*x*_ is the dry weight of the tissue x (in g).

The quantity of element *n* taken up (Up) by the plant (expressed in mg) was calculated as:


$${\left(U\,p\right)}_{n}=Q\,t\,y_{n\,\left(p\,l\,a\,n\,t\right)}\,\left(t\,d_{n}\right)-Q\,t\,y_{n\,\left(p\,l\,a\,n\,t\right)}\,\left(t\,0\right)$$

where Qty_*n(plant)*_ (td_*n*_) is the quantity of the element n in the plant at td_*n*_ (in mg) and Qty_*n(plant)*_ (t0) is the quantity of the element “n” in the plant at t0 (in mg).

The percentage of element allocated to a tissue *x*, hereafter called Allocation (A) (expressed in %) was calculated as:


$$A_{x}=\frac{Q\,t\,y_{n\,\left(x\right)}}{Q\,t\,y_{n\,\left(p\,l\,a\,n\,t\right)}}\,\text{×}\,100$$

where Qty_*n(x)*_ is the quantity of the element *n* in the tissue *x* (in mg) and Qty_*n(plant)*_ is the quantity of the element *n* in the plant (in mg).

The quantity of element *n* that was remobilized during the deficiency period (R) (expressed in mg) was calculated by mass balance (i.e., net remobilization) in the following manner:


$$R\,n=Q\,t\,y_{n\,\left(x\right)}\,\left(t\,d_{n}\right)-Q\,t\,y_{n\,\left(x\right)}\,\left(t\,0\right)$$

where Qty_*n(x)*_ (td_*n*_) is the quantity of the element *n* in the tissue *x* at td_*n*_ (in mg) and Qty_*n(x)*_ (t0) is the quantity of the element *n* in the tissue *x* at t0 (in mg).

Statistical analyses were performed with R software^1^. Comparisons were performed between t0 and td_*n*_ for control and deficient plants and between control and deficient plants at td_*n*_ and tm_*n*_. To analyze the effects of different deficiencies on measured and calculated variables, *t-*tests were carried out *via* bootstrap tests using boot.test() (package boot and MKinfer). The effect was considered significant at a *p*-value inferior to 0.05.

## Results

### Essential and Beneficial Element Accumulation During Pea Growth

**FIGURE 2:**
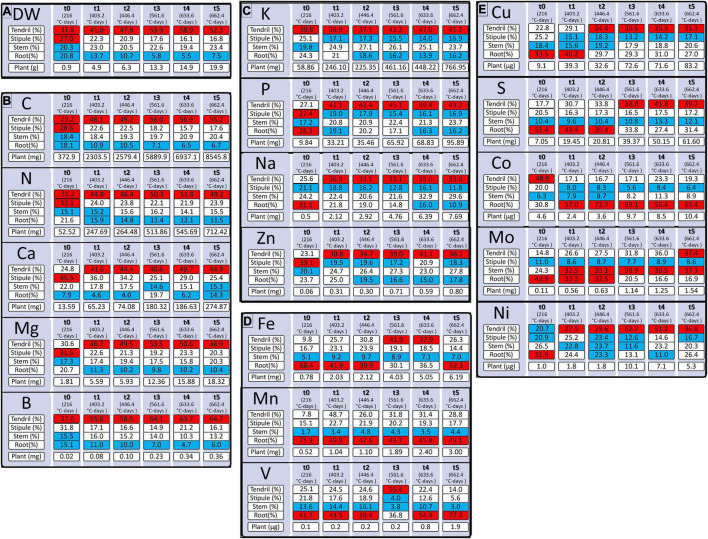
Accumulation of dry weight [DW, **(A)**] and quantity of mineral elements **(B–E)** during pea vegetative growth from 216 to 662.4 degree-days (°C-days). Mineral elements are grouped by the quantity needed for pea plant growth. Mineral elements are N, Nitrogen; K, Potassium; Ca, Calcium, P, Phosphorus; S, Sulfur; Mg, Magnesium; Fe, Iron; Mn, Manganese; Mo, Molybdenum; Na, Sodium; Zn, Zinc; B, Bore; Cu, Copper; Ni, Nickel; V, Vanadium; Co, Cobalt. Tendril, Stipule, Stem, Root (%) correspond to the percentages of the element allocated to the organ concerning the total element quantity in the plant. “Plant” refers to the total quantity of an element in the plant in mg or μg according to the element considered. Red boxes correspond to tissues where the element is allocated in the highest quantity and blue boxes correspond to tissues where the element is the least allocated. Values (n varied from 4 to 5).

The allocation of dry matter (DW) to the shoot tissues increased progressively with time at the expense of the allocation to the roots (Figure 2A). Carbon accumulation in the plant and its allocations (Figure 2B) to the different tissues followed approximatively the same pattern (Supplementary Figure 1).

The highest macro-nutrients were N and K while amongst micro-nutrients Fe, Mn, and Mo were those with the highest accumulation in the vegetative tissues of the plant (Figure 2). The different elements were mostly allocated to the shoots, except for S, Fe, and Mn at t0 (15 days), Fe at t5 (32 days), Co except at t0 (15 days), and V from t4 (45 days) that were preferentially allocated to the roots. Between t1 (403°C-days) and t5 (662°C-days), all elements in the shoots that were divided into the stems, stipules, and tendrils, were mostly allocated to the tendrils except for Mo that was, for the most part, allocated to the stem. The higher quantity of element in tendrils can be explained by the preferential allocation of dry weight to this tissue during pea growth (Figure 2A), and even if lower concentrations of N, Mg, P, S, Ca, V, Mn, Fe, and Cu were measured in the tendrils regarding stipules (Supplementary Figure 1 and Supplementary Table 1).

Finally, based on the patterns of element allocation within the plant throughout its development, three groups of elements were identified. The first group, composed of N, Ca, Mg, B, and C, was characterized by the allocation of elements preferentially to the tendrils and a lesser extent to the roots (Figure 2B). The second group, composed of P, K, Zn, and Na, was characterized by the allocation of elements primarily to the tendrils and to a lesser extent to the roots and/or stipules according to the age of the plant (Figure 2C). Finally, the third group, composed of Fe, Mn, and V, was characterized by the allocation of elements primarily to the roots and to a lesser extent to the stems (Figure 2D). However, S, Mo, Cu, and Co cannot be classified into one of these three groups (Figure 2E) as their accumulation pattern among tissues varied throughout plant growth.

### Remobilization Strategies Differ Among Deprived Elements

**FIGURE 3:**
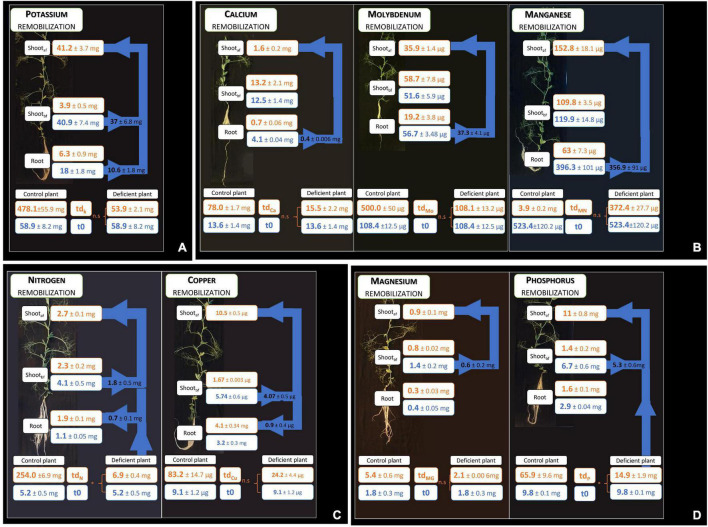
Remobilization of elements after element deprivation. The compartments consider root and shoot growth before deprivation (shoot_*bf*_) and shoot growth under deprivation (shoot_*af*_); a non-significant uptake during deprivation is characterized by n.s, whereas an uptake during deprivation is characterized by *. Arrows represent the quantity of element remobilized from grown tissues to growing tissues. Four groups of remobilizations are observed in the pea according to sink and source organs **(A–D)**. Values ± SD (n varied from 4 to 5).

In this study, 13 elemental deprivations were applied to pea plants at the vegetative stage. Each deprivation was continued until the cessation of growth was observed. The negative impact of macro-nutrient deprivation during vegetative growth on biomass production was observed before that of micro-element deprivation, except in the cases of Mo and Fe deficiencies (Figure 1B): the first deficiency was established after 14 days (14 leaves stage) of nutrient removal in the case of N deficiency, followed by Mg deficiency (15 days, 15 leaves stage), S, Ca and Mo deficiencies (16 days, 16 leaves stage), P deficiency (24 days, 20 leaves stage), Fe deficiency (28 days, 22 leaves stage), K deficiency (29 days, 22 leaves stage), and Cu deficiency (32 days, 24 leaves stage) (Figure 1B). For Ni, B, Mn, and Zn deprivations, no cessation of growth was observed after 42 days (29 leaves stage) of nutrient removal (Figure 1B). Despite the nutrient removal from the nutrient solution, significant nutrient uptakes were observed for N, P, Zn, S, Fe, B, and Ni under their elemental deprivation (Supplementary Table 2), probably because of the presence of trace elements in the solution. However, their uptake under deprivation was reduced by 73.3, 78.8, 76.9, 44.2, 74.6, 61.2, and 58.9%, respectively, compared with control plants. The net remobilization of these elements was thus not quantified, even if remobilization was noticeable in the cases of N and P deprivations (Figures 3B,C). Indeed, a significant decrease in the quantity of these two elements was observed in the shoots present before deprivation (Shootbf).

For the other nutrients, four main types of remobilizations, classified according to either a sink behavior (net increase of element content) or a source behavior (net decrease of element content), were observed (Figure 3). K was the only element remobilized from both root and shoot grown before deprivation to the shoot grown during deprivation (Figure 3A). Moreover, the percent of remobilization of K measured in shoot growth before deprivation was higher (91.1%) than the percent of remobilization of K measured in the root (58.9%). N and Cu were remobilized from the shoots present before deprivation to both shoots growing under deprivation and to the roots, although this measurement was biased by a residual N uptake that may have occurred during N deprivation (Figure 3C). Under Cu deprivation, a higher quantity of Cu was remobilized from shoots present before deprivation to shoots growing under deprivation (78%) compared with the Cu remobilized from roots to shoots growing under deprivation (22%). On the other hand, Mg and P were remobilized from the shoots present before deprivation to shoots growing under deprivation only, but not to the roots (Figure 3D). In addition, the percent of remobilized P is higher (79.1%) than the percent of remobilized Mg (42.8%), with approximately twice as much percent of P as the percent of Mg removed from shoots present before deprivation to shoots growing under deprivation. The last type of remobilization concerned the Ca, Mo, and Mn that were remobilized from roots to shoots with, respectively, 83, 66, and 84% of Ca, Mo, and Mn remobilized (Figure 3B).

### Essential Element Interactions During Nutrient Deficiencies

**FIGURE 4:**
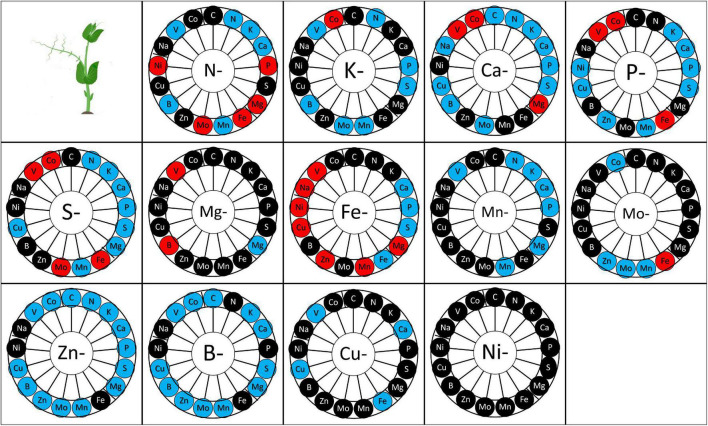
Mineral element quantity in the plant according to the elementary deprivation considered. Deprivations include N-, Nitrogen deprivation; K-, Potassium deprivation; Ca-, Calcium deprivation; P-, Phosphorus deprivation; S-, Sulfur deprivation; Mg-, Magnesium deprivation; Fe-, Iron deprivation; Mn-, Manganese deprivation; Mo-, Molybdenum deprivation; Zn-, Zinc deprivation; B-, Bore deprivation; Cu-, Copper deprivation; Ni-, Nickel deprivation. Elements measured are C, Carbon; N, Nitrogen; K, Potassium; Ca, Calcium; P, Phosphorus; S, Sulfur; Mg, Magnesium; Fe, Iron; Mn, Manganese; Mo, Molybdenum; Na, Sodium; Zn, Zinc; B, Bore; Cu, Copper; Ni, Nickel; V, Vanadium; Co, Cobalt. Elements in red are those whose quantity increased as a result of deprivation, elements in blue are those whose quantity decreased and elements in black are those whose quantity was maintained after deprivation. Values (n varied from 4 to 5).

To obtain better insight into the interactions among elements, their quantifications were performed at the end of each nutrient deprivation period and summarized in Figure 4. Whatever the nutrient deprivation, a lower accumulation of the deprived element was observed, except in the case of Ni deprivation, in which Ni accumulation did not change (Figure 4 and Supplementary Table 3). For example, nitrate deprivation significantly reduced N quantity (by about 73%) in the deprived plant in comparison to the control plant. These results thus confirm the establishment of nutrient deficiency except in the case of Ni.

Overall, the deprivation of one nutrient affects the ionome composition of the plant largely (Figure 4) except for Ni deprivation. Mn, Zn, Cu, and B deprivation negatively impacted or have no impact on the uptake of other elements, while the other deprivations revealed both positive, negative, or neutral impacts on the accumulation of other elements (Figure 4). Some nutrients appeared to be reciprocally linked, i.e., deprivation of element “a” decreased the quantity of element “b” and inversely. For instance, N deprivation reduced K, Ca, and Mn accumulation in the plant, and K, Ca and Mn deprivation likewise reduced N accumulation in the plant, in comparison with the control plants. The same type of link was observed between S and (P, K, Ca, B), between P and (S, K, Ca, Mn, Fe), between K and (N, S, P, B, Mn, Zn), between Ca and (N, S, P, P, B, Cu), between Mo and (B, Zn), between Fe and P, between Zn and (P, K, Mo), between B and (S, K, Zn), between Cu and Ca, and between Mn and (N, P, K).

In contrast, some elements were antagonistically linked, i.e., the deprivation of element “a” reduced the quantity of element “b” but the deprivation of element “b” increased the quantity of element “a” in comparison to control plants. This was the case for Fe and S, B and Mg, Cu and Fe.

On the other hand, some elements were non-reciprocally linked, i.e., the deprivation of element “a” modifies the quantity of element “b”, but the deprivation of element “b” does not modify the quantity of element “a”. This was the case of N with P, B, Fe, Mo; K with Mo; Ca with K, Mg, Mo; P with Cu; S with Cu, Mn, Mg, N and Mo; Fe with Ca, Mn, and Mg; Mn with Ca and Mg; Mo with Fe and Mn; Zn with S, Fe, Ca, Cu, Mn, and Mg; B with Cu, Mn, and Mo.

### Transitory Nutrient Deficiency Impacted Seed Yield Components and Seed Mineral Composition

**FIGURE 5:**
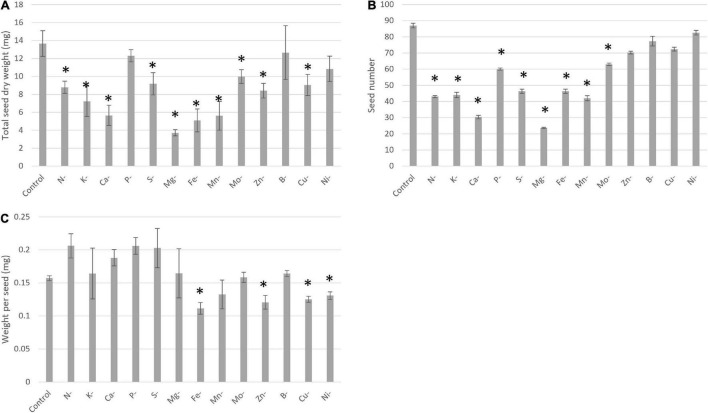
Yield components under control and deprived conditions, seed dry weight **(A)**, seed number **(B)**, and one seed dry weight **(C)**. Conditions are: Control; N-, Nitrogen deprivation; K-, Potassium deprivation; Ca-, Calcium deprivation; P-, Phosphorus deprivation; S-, Sulfur deprivation; Mg-, Magnesium deprivation; Fe-, Iron deprivation; Mn-, Manganese deprivation; Mo-, Molybdenum deprivation; Zn-, Zinc deprivation; B-, Bore deprivation; Cu-, Copper deprivation; Ni-, Nickel deprivation. All deprivations occurred during vegetative growth for a duration that depended on the element considered. *Corresponds to deprivations that present a significant difference with the Control. Values ± SD (*n* = 4).

In our experiment, the transitory elemental deprivation period to which the pea plants were subjected during the vegetative stage was followed by a recovery period until plant physiological maturity. After ten transitory deprivations during vegetative growth, yield components such as seed dry weight, seed number, and/or weight per seed were significantly affected (Figure 5). A lower seed dry weight was observed for N, K, Ca, S, Mg, Fe, Mn, Mo, Zn, and Cu deficiencies (Figure 5A). This lower seed dry weight was linked to a lower seed number, except for Zn and Cu deprivations (Figure 5B) for which the lower seed dry weight was linked to a lower weight per seed (Figure 5C). Moreover, the more deleterious deprivation effects on yield components were found for Ca, Mg, Fe, Mn with total seed weight and a seed number reduced by at least 50% compared with the control plants.

**FIGURE 6:**
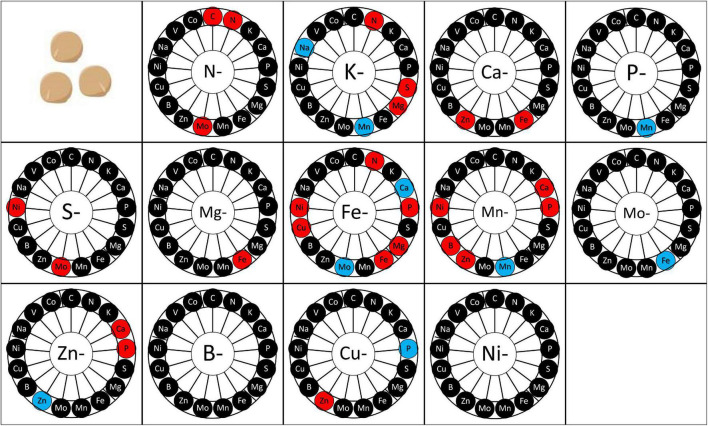
Mineral element concentration in seeds according to the elementary deprivation considered. Deprivations included N-, Nitrogen deprivation; K-, Potassium deprivation; Ca-, Calcium deprivation; P-, Phosphorus deprivation; S-, Sulfur deprivation; Mg-, Magnesium deprivation; Fe-, Iron deprivation; Mn-, Manganese deprivation; Mo-, Molybdenum deprivation; Zn-, Zinc deprivation; B-, Bore deprivation; Cu-, Copper deprivation; Ni-, Nickel deprivation. Elements measured are C, Carbon; N, Nitrogen; K, Potassium; Ca, Calcium; P, Phosphorus; S, Sulfur; Mg, Magnesium; Fe, Iron; Mn, Manganese; Mo, Molybdenum; Na, Sodium; Zn, Zinc; B, Bore; Cu, Copper; Ni, Nickel; V, Vanadium; Co, Cobalt. Elements in red are elements whose concentration increased after deprivation, elements in blue are elements whose concentration decreased after deprivation, and elements in black are those whose concentration was maintained after deprivation. Values (*n* = 4).

At physiological maturity, the concentration of each element was measured in seeds (Figure 6 and Supplementary Table 4). B and Ni deprivation did not impact the mineral composition of seeds. Conversely, a transient Zn or Mn deprivation increased some element concentrations in mature seeds in comparison to control plants. Interestingly, only four transitory deprivations (N, Fe, Mn, and Zn) impacted the concentration of the deprived element at maturity (Figure 6). In some cases, transitory deprivation enhanced the concentration of the deprived element in seeds. It was the case for N deprivation and Fe deprivation. Conversely, transitory deprivation of Zn induced a decrease in Zn quantity in seeds at maturity in comparison to control plants. The transitory deprivation that most modified the mineral composition of seeds was the Fe deprivation, increasing the N, Mg, Cu, and Fe concentrations and decreasing Mo and Ca concentrations.

## Discussion

In our study, the 13 mineral nutrient deprivations applied to pea plants allowed us to have a better knowledge about their uptake, their allocations in plant compartments, their remobilization between source and sink tissues, and the interactions among elements for these processes. Moreover, the effect of mineral nutrient deprivation on yield components (seed number and seed dry weight) and seed quality (mineral composition), have been characterized.

### Nutrient Allocation Within Plant Under Non-stressful Conditions

Under non-limiting nutrient conditions and more broadly non-stressful conditions, the majority of plant biomass and carbon was allocated to shoot organs and notably to tendrils for this pea *afila* variety (Figure 2). This is congruent with non-limiting conditions concerning water and nutrients that enable the necessary nutrition for plant growth. Indeed, under non-limiting conditions, root growth is not privileged since all necessary nutrients and water are available for the root system (Ma et al., 2001; López-Bucio et al., 2003). Moreover, a higher allocation to tendrils compared with stipules may be correlated with the low surface of stipules. This can be explained by a higher xylem flux in tendrils that present a higher exchange surface than stipules, *via* a higher transpiratory rate in tendrils (Salon and Munier-Jolain, 2010). For the majority of elements (N, Mg, P, S, Ca, Fe, and Cu), the higher quantity of elements in tendrils regarding stipules was linked to higher biomass despite lower concentrations. In this way, several elements were accumulated preferentially in the tendrils, notably N and that could be linked to C accumulation in biomass production. However, some elements (S, Fe, Mn, Co, and V) were preferentially allocated to the roots at different growing stages. This was previously observed in other pea cultivars where higher proportions of Cu and Cd (Landberg and Greger, 1994), Mn and Fe (Nenova, 2008) were allocated to the root system. Another factor that may explain element partitioning in different organs is the relationship with others. Indeed, elements are linked by common physiological processes, either *via* a common transporter (Gassmann et al., 1996; Dudev and Lim, 2004; Pilon et al., 2009), or osmotic and acido-basic equilibrium maintenance (Kirkby and Knight, 1977; Sorin et al., 2015), or their role in a common metabolic pathway (Kuper et al., 2004; Schwarz and Mendel, 2006). For instance, Fe and Mn are transported by the same transporters (IRT1, Iron-Responsive Transporter 1; Ys-YSL, Yellow-Stripe 1-like; ZIP, ZRT-IRT_like Proteins) and present in the same tissue for the most (Pilon et al., 2009). In addition, K and Na present the same pattern of accumulation, an observation that may be explained by their role in potential osmotic maintenance and by some common transporter (Schachtman and Liu, 1999). Thus, allocation to different organs seems to depend on the biochemical functions of a group of nutrients and their transport, which may be common. In our study, plants were not inoculated with rhizobia and were not able to form any nodule in the root system. This leads to an N uptake that is only based on a mineral uptake of N. Because N fixation can induce changes in the pattern of nutrient uptake, promoting an alkaline nutrient uptake unlike acidic uptake pattern observed in nitrate-supplied plants (Van Beusichem, 1981), we can suppose that our results could not be directly transposed to plants grown without mineral N and under dinitrogen-fixing conditions.

Mineral allocation on different plant tissues depends on the quantity of the element taken up and on its storage in the plant (Malagoli, 2005; Gojon et al., 2009; Pilon et al., 2009; White, 2012). To enable optimal growth and development during the reproductive phase or under abiotic stresses, mineral elements are then remobilized from old tissues to growing tissues (Malagoli, 2005; Masclaux-Daubresse et al., 2008). Indeed, some stresses like water deficiency reduce mineral element availabilities in the soil for plants (Wiersum, 1958). Thus, the remobilization of mineral elements under limiting availability can allow plants to maintain their growth during a given period (Masclaux-Daubresse et al., 2008).

### Four Patterns of Remobilization Identified Under Nutrient Deficiency

In our study, in the case of seven nutrient deprivations, a significant uptake was observed after the control solution was replaced by an elementary deprived solution. This observation applies to the N, P, Zn, S, Fe, B, and Ni deficiencies. In pea plants, four patterns of remobilization that depend on the source and sink organs were highlighted (Figure 3). Indeed, the remobilization of a deprived element may start in an old shoot and/or root and move to a young shoot and/or root. This first pattern of remobilization concerned only K, with remobilization from all organs present before K deprivation (Figure 3A). Indeed, K was remobilized from roots and old shoots to young shoots. In the second and third patterns, elements were remobilized from old shoots to young shoots and/or roots (Figures 3B,C). These two types of remobilizations concerned N, Cu, Mg, and P. It is interesting to observe the same pattern of remobilization in the cases of N and Cu in the pea plant, which could be linked to the high induction of Cu remobilization by an N deficiency (Hill and Stobbe, 1978). Moreover, as in the case of N remobilization, there is a correlation between leaf senescence and Cu remobilization (Himelblau and Amasino, 2001). Thus, under N and Cu deprivation, these two elements would be, respectively, remobilized from old shoots *via* the senescence process to meet the needs of growing shoots and roots.

On the other hand, Mg and P were remobilized from old shoots to growing shoots, but not to the roots (Figure 3C). However, Mg deficiency was established faster than P deficiency, after 15 and 24 days, respectively. This difference could be explained by the lower efficiency of mobilization of Mg in comparison to the efficiency of mobilization of P, yet observed for legume plants (Hocking and Pate, 1977). Lastly, the fourth strategy of remobilization that we highlighted concerned Ca, Mn, and Mo, for which remobilization was based solely on roots, not on shoots (Figure 3D). It could be explained by the low phloem mobility of these elements and the potential remobilization of these elements from the root *via* the xylem (Nable and Loneragan, 1984; Dayod et al., 2010; White, 2012; Maillard et al., 2015). Indeed, this process has been observed in soybeans (*Glycine max*) and in green beans (*Phaseolus vulgaris*) for Ca (Biddulph et al., 1959; Mauk and Noodén, 1992). Moreover, the lack of Ca and Mn mobility is not species-dependent, unlike Mo. Indeed, Mo exhibits phloem mobility in different species, like wheat (*Triticum sativum*) although a lack of mobility has been demonstrated in pea plants (White, 2012; Maillard et al., 2015). Thus, the remobilization of these Ca, Mn, and Mo is nonetheless observed from roots to shoots, which suggests a xylem transport to ensure the needs of the growing shoots (Maillard et al., 2015). In this way, the lack of Mo shoot remobilization may explain the establishment of deficiency of this micro-nutrient after the same duration as that observed in Ca deficiency establishment. Thus, the establishment of mineral element deficiency depends not only on element quantity required but also on the remobilization efficiency of this element. Thus, because of the difference in remobilization efficiency under element deprivation, a specific element deprivation can be more limiting due to its lower remobilization.

### Plant Nutrition: A Complex Network of Mineral Elements

Our study highlighted interactions between elements in the pea plant under 13 mineral nutrient deprivation conditions (Figure 4). The uptake of an element may be antagonist or synergic with other elements, depending in some cases on the acido-basic and osmotic equilibrium. To maintain the acido-basic plant equilibrium, a reduction in the quantity of an acid element may induce a reduction in a basic element (Kirkby and Knight, 1977; Sorin et al., 2015). For instance, N deprivation induces a reduction in K and Ca uptake that could compensate for the reduction in the quantity of anion (Kirkby and Knight, 1977). Moreover, the reduction of nutrient quantity induced by an elementary deprivation may be due to the involvement of the deprived element in the biological process. Indeed, in the case of Mo cofactor biosynthesis, Zn and Cu are involved in its biosynthesis. For peas, a decrease of Mo quantity is observed under Zn deprivation (Kuper et al., 2004; Schwarz and Mendel, 2006; Llamas et al., 2011). That can be due to the lower consumption of Mo for Mo cofactor biosynthesis. On the other hand, some nutrients are characterized by antagonistic uptake; this is the case for S and Mo. A deprivation of S induces an increased uptake of Mo in the pea plant as observed for rapeseed (Maillard, 2016).

Some mineral deprivation solely induced a reduction in the quantity of another element, in addition to the nutrient-deprived. This wholly negative impact on other elements concerns four micro-nutrients studied here, namely Mn, Zn, Cu, and B. However, in the cases of other nutrient deprivation, positive and negative impacts were observed on the quantities of other elements. Among these elements, Fe deprivation was the one that resulted in the most frequent increases in the quantities of other elements. This enhancement of element uptake could be explained by a higher expression of non-specific transporter induced by deprivation. Indeed, the divalent metal co-transporter IRT1 (Iron-Regulated Transporter 1) enables the uptake of Ni, Cu, Mn, Zn, and Fe and is induced by Fe deficiency (Pilon et al., 2009). In addition, Ys-YSL (Yellow-Stripe 1-like) transports Cu, Mn, Zn and Fe, and ZIP (ZRT-IRT_like Proteins) transports Mn, Zn, and Fe (Krämer et al., 2007). Thus, under Fe deprivation, competition for uptake of other cations is reduced even if the activity of the transporter is enhanced. In this way, Ni, Cu, Mn, and Zn are more easily taken up from the soil by the root regarding non-limiting Fe condition. However, Fe deprivation induces a reduction in other elements including P, S, and Ca, all of which play important roles in plant growth and development. Indeed, S is a major component of protein with 50% of the total S quantity integrated in protein in rapeseed plant (Maathuis, 2009), while P and Ca are important for membrane stability (Maathuis, 2009), and Ca is important in the regulation of Krebs cycle and osmotic equilibrium (Maathuis, 2009).

If some relations among mineral elements seem to be generic, being shared by several species and genotypes, we need to keep in mind that strong genotypic effects and genotype × environment interactions can modulate correlations among elements, as demonstrated in Arabidopsis (Baxter et al., 2012) or maize (Stich et al., 2020).

### Toward Strategies to Improve Seed Quality Through Mineral Nutrition

Transitory deficiencies have an impact on plant ionome during vegetative growth that can be conserved or not during reproductive growth. Indeed, mineral nutrient deficiencies can negatively impact seed quantity and mineral composition (McGrath and Zhao, 1996). For instance, the negative impact of S deficiency has been observed in canola plants that require an important quantity of S during its crop cycle as it induced a delay of flowering and maturity and a production of smaller and poorly filled pods (Grant and Bailey, 1993). For canola, a reduction of seed yield is also observed after B deficiency, as B represents another mineral element important for its development (Nyborg and Hoyt, 1970). Moreover, like for canola, rapeseeds need an important quantity of S and present a reduction of seed quality under S deficiency (D’Hooghe et al., 2014). The reduction of rapeseed seed quality is associated with a decrease of seed viability and a reduction of accumulation of protein-rich in S. Our study also deepens our understanding of the effects of transitory deficiency on pea seed yield and composition. Indeed, our results revealed an impact of all deprivations, except deprivation in B and Ni, during vegetative growth on seed yield and/or seed composition at maturity. In pea, a negative impact of mineral deprivation was observed for N, S, P, Ca, Mg, and Fe, Mn with a reduction of seed number that was correlated with a decrease of seed biomass for N, S, Ca, Mg, and Fe. The most important deleterious deprivation impacts on seed yield were observed for Ca, Mg, Mn, and Fe deficiencies (Figure 5). However, the more negative impact of these deprivations on yield regarding to N deprivation in pea could be due to different durations of recovery. Indeed, the recovery period allowed for most of the plants to offset the mineral element quantity missing at the end of the deprivation period in comparison to control plants (Figure 6). Only four transitory deprivations revealed an impact on the concentration of the deprived element in seeds. Two cases revealed a lower concentration after Mn and Zn deprivation; two revealed a higher concentration, i.e., N and Fe. The lower concentration of Mn and Zn could be linked either to a lower potential of remobilization of these two elements under non-limiting conditions (Sankaran and Grusak, 2014) or/and to a lower quantity of these two elements after deprivation. We can conclude that following N and Fe deprivation, the uptake and remobilization of these elements to seeds seemed to increase. In the case of transitory N deprivation, seed N and Mo concentrations increased despite a reduction of seed quantity and biomass. So, mineral deprivation can antagonistically impact seed yield and mineral composition, i.e., produce seeds richer in N and Mo even if yields are reduced. Moreover, Fe deprivation during vegetative growth induced a higher concentration of N, Mg, P, Cu, and Fe, but also of Ni. However, the increase in Ni concentration does not represent an improvement in seed quality, nor does a decrease in Mo and Ca concentration. So, an improvement of knowledge on the ionomic imprint induced by different deprivations could enable to ensure yield and seed mineral composition thanks to appropriate cultural practice. Thus, an enhancement of nutrient uptake or remobilization after a nutrient deficiency period (*via* fine control of fertilization or bio-inoculation) would improve seed quality.

## Conclusion

The present study deepens our understanding of mineral nutrient uptake, storage, and remobilization in the pea plant. In this species, we have been able to classify elements in order of importance: N, K, Ca, P, S, Mg, Fe, Mn, Mo, Zn, Cu, and Ni. Moreover, beneficial elements are also required in different quantities, with a higher quantity of Na required compared to V and Co. Differences in the allocation of these elements observed in two leaf tissues (stipules and tendrils) have been characterized. For eight mineral nutrients, the remobilization has been characterized into four different groups that depended on relations between sink and source organs during vegetative growth. Indeed, K was remobilized from roots and shoots to shoots, N and Cu from shoots to shoots and roots, Mg and P from shoots to shoots; and Ca, Mo, and Mn from roots to shoots. The study of the pea ionome allowed us to better understand the effects of a deficiency on the nutritional status of the plant and the time needed to the establishment of deficiency in a single cultivar of pea (cv. Kayanne) grown under non-N_2_-fixing conditions. Moreover, our study of the seed ionome highlighted a possible positive impact of transitory deficiency during vegetative growth on seed quality *via* the increase in the uptake of several nutrients. A detailed understanding of deprivation impacts may enable users to fine-tune the use of fertilizers when essential for optimal plant growth in soil under conditions of simple mineral nutrient deprivation, or under sub-optimal environmental conditions such as water stress.

## Data Availability Statement

The original contributions presented in the study are included in the article/Supplementary Material, further inquiries can be directed to the corresponding author.

## Author Contributions

AO, CS, and MP conceived the project. CJ, MF, VD, and MP performed the experiments. CJ analyzed the data. CJ, MF, VD, CS, AO, and MP contributed to the writing and approved the final manuscript. All authors contributed to the article and approved the submitted version.

## Conflict of Interest

The authors declare that the research was conducted in the absence of any commercial or financial relationships that could be construed as a potential conflict of interest.

## Publisher’s Note

All claims expressed in this article are solely those of the authors and do not necessarily represent those of their affiliated organizations, or those of the publisher, the editors and the reviewers. Any product that may be evaluated in this article, or claim that may be made by its manufacturer, is not guaranteed or endorsed by the publisher.

## Footnotes

1 https://www.r-project.org/, v.3.5.2
