# Supplementary material for: Transient Nutrient Deficiencies in Pea: Consequences on Nutrient Uptake, Remobilization, and Seed Quality
Source: Front Plant Sci. 2021 Dec 23;12:785221. doi: 10.3389/fpls.2021.785221 (PMC8733391; doi:10.3389/fpls.2021.785221)
Supplement: Supplementary file 2 [file Data_Sheet_2.docx]

Supplementary Figures

**Figure S1**


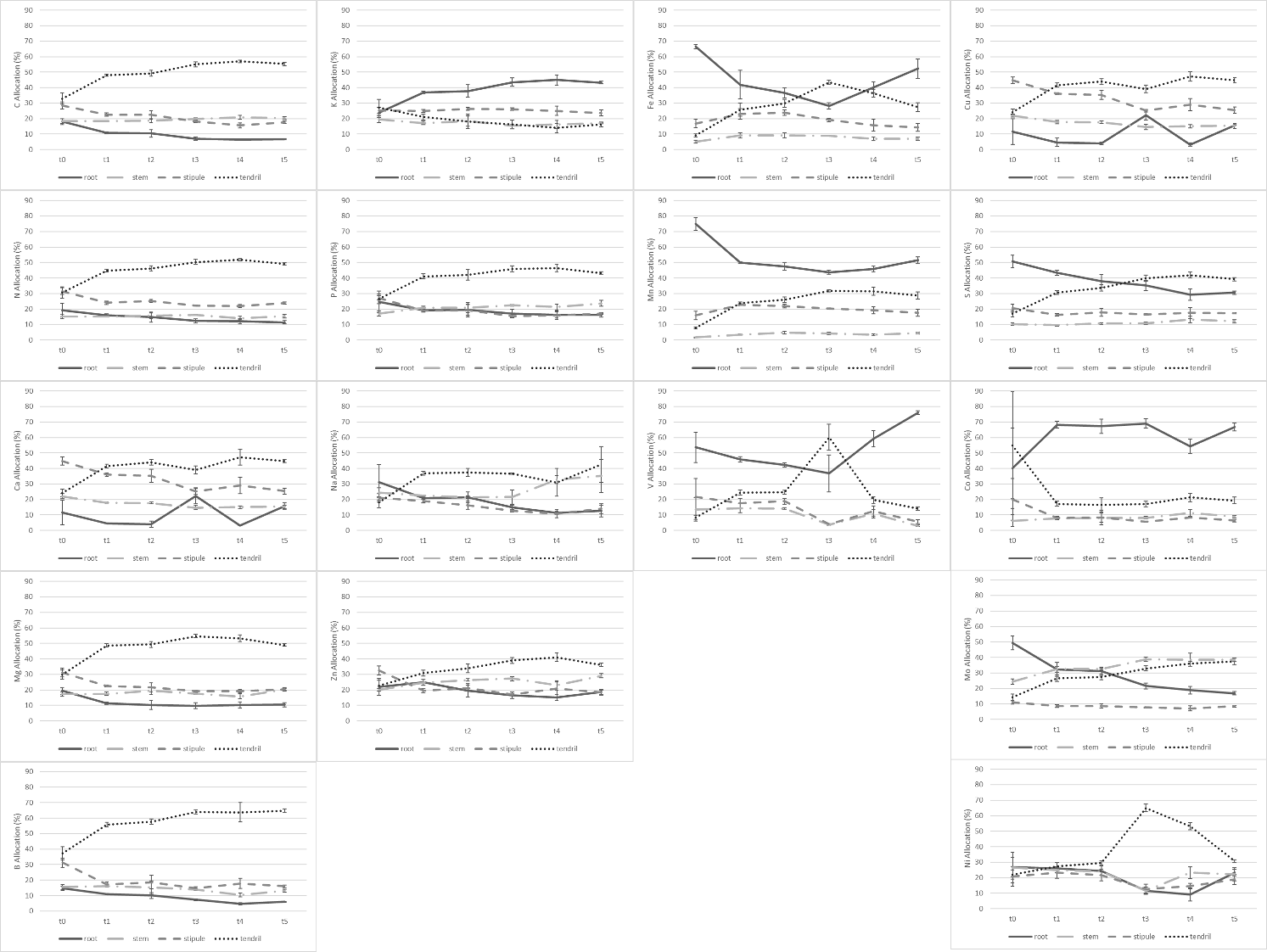


**Supplementary Figure S1. Allocation of nutrients (%) in four pea tissues (tendril, stipule, stem and root) during pea vegetative growth** from 216 to 662.4 C-days. Elements are N, Nitrogen; K, Potassium; Ca, Calcium. P, Phosphorus; S, Sulphur; Mg, Magnesium; Fe, Iron; Mn, Manganese; Mo, Molybdenum; Na, Sodium; Zn, Zinc; B, Bore; Cu, Copper; Ni, Nickel; V, Vanadium; Co, Cobalt.
